# The NADase CD38 is a central regulator in gouty inflammation and a novel druggable therapeutic target

**DOI:** 10.1007/s00011-024-01863-y

**Published:** 2024-03-16

**Authors:** Paulo Gil Alabarse, Patricia Oliveira, Huaping Qin, Tiffany Yan, Marie Migaud, Robert Terkeltaub, Ru Liu-Bryan

**Affiliations:** 1 VA San Diego Healthcare System, 111K, 3350 La Jolla Village Drive, San Diego, CA 92161 USA; 2 University of California San Diego, La Jolla, San Diego, CA USA; 3 Present Address: The Janssen Pharmaceutical Companies of Johnson & Johnson, La Jolla, San Diego, CA USA; 4 Present Address: Gritstone Bio, Emeryville, CA USA; 5 Department of Pharmacology, F. Whiddon College of Medicine, Mitchell Cancer Institute, University of South Alabama, Mobile, AL 36604 USA

**Keywords:** CD38, NAD^+^, Macrophages, Gout, Inflammation

## Abstract

**Objectives:**

Cellular NAD^+^ declines in inflammatory states associated with increased activity of the leukocyte-expressed NADase CD38. In this study, we tested the potential role of therapeutically targeting CD38 and NAD^+^ in gout.

**Methods:**

We studied cultured mouse wild type and CD38 knockout (KO) murine bone marrow derived macrophages (BMDMs) stimulated by monosodium urate (MSU) crystals and used the air pouch gouty inflammation model.

**Results:**

MSU crystals induced CD38 in BMDMs in vitro, associated with NAD^+^ depletion, and IL-1β and CXCL1 release, effects reversed by pharmacologic CD38 inhibitors (apigenin, 78c). Mouse air pouch inflammatory responses to MSU crystals were blunted by CD38 KO and apigenin. Pharmacologic CD38 inhibition suppressed MSU crystal-induced NLRP3 inflammasome activation and increased anti-inflammatory SIRT3–SOD2 activity in macrophages. BMDM RNA-seq analysis of differentially expressed genes (DEGs) revealed CD38 to control multiple MSU crystal-modulated inflammation pathways. Top DEGs included the circadian rhythm modulator *GRP176*, and the metalloreductase *STEAP4* that mediates iron homeostasis, and promotes oxidative stress and NF-κB activation when it is overexpressed.

**Conclusions:**

CD38 and NAD^+^ depletion are druggable targets controlling the MSU crystal- induced inflammation program. Targeting CD38 and NAD^+^ are potentially novel selective molecular approaches to limit gouty arthritis.

**Supplementary Information:**

The online version contains supplementary material available at 10.1007/s00011-024-01863-y.

## Introduction

In gout, flares of severely painful inflammatory arthritis intersect with metabolism, and macrophage activation [1–3]. Gout represents a major public health problem, and severe flares commonly lead to physical incapacity, emergency department visits, and hospitalization [1]. Moreover, recent gout flare is a major risk factor for non-lethal and lethal myocardial infarction and stroke as subsequent cardiovascular events [4].

Gout flares are principally prevented and treated with nonselective, frequently toxic drugs (colchicine, NSAIDs, and corticosteroids). Gout flares are a phenotype of the acute inflammatory response to deposits of monosodium urate (MSU) crystals. Interaction of MSU crystals with resident cells such as macrophages induces acute inflammation in large part via induction of NF-κB and NLRP3 inflammasome activation, and consequent release of inflammatory cytokines [5–7]. Evolving strategies to treat flares with biologic agents that selectively target IL-1β and the NLRP3 inflammasome have illustrated the impact of the current understanding of the molecular mechanisms involved in gouty inflammation [8].

This study focuses on nicotinamide adenine dinucleotide (NAD^+^) metabolism as a potentially selective molecular target niche for gouty inflammation. NAD^+^ is a necessary cofactor and key metabolite in pathways involved in cellular energy homeostasis and adaptive responses of cells to bioenergetic stressors including inflammation and aging [9, 10]. Intracellular NAD^+^ levels are significantly affected by environmental stimuli (9, 10) and diverse cell stressors [9, 10]. For example, intracellular depletion of (NAD^+^) is promoted by aging and inflammatory diseases [9, 10]. In turn, NAD^+^ depletion promotes aging-related tissue damage and inflammation, partly mediated by priming of NLRP3 inflammasome activation in macrophages [11, 12]. As such, boosting cellular NAD^+^ levels are an emerging, broadly investigated, and selective molecular approach to diseases of aging and inflammation [9, 10].

Changes in cellular NAD^+^ levels are related to NAD^+^ biosynthesis and/or degradation. Most NAD^+^ is synthesized through the salvage pathway from nicotinamide (NAM), the by-product of NAD^+^ degradation [9, 10], which can be promoted by Cluster of differentiation 38 (CD38) [9, 10, 13]. CD38 has both NAD^+^ glycohydrolase and ADP-ribosyl cyclase activities, and thereby catalyses production of ADP-ribose (ADPR), cADPR, and NAM [9, 10, 13]. That said, CD38 NAD^+^ glycohydrolase “NADase” function represents > 90% of CD38 enzyme activity [10, 13]. CD38 is the principal NADase in mammalian tissues [10, 13], evidenced by tissue NAD^+^ levels being 10- to 20-fold higher in CD38 knockout (KO) compared to wild type (WT) mice [13]. In addition, overall NADase activity is attenuated in CD38KO mice [13].

CD38 is ubiquitously expressed in all immune cells including macrophages [14–16]. CD38 expression increases in diseases associated with inflammatory conditions [14–16]. A recent study showed that MSU crystals up-regulate CD38 and reduce intracellular NAD^+^ levels in human and mouse macrophages in vitro [17]. NAD^+^ degradation by CD38 is strongly linked to reduced activities of NAD^+^-dependent protein deacetylase family of sirtuins (SIRTs) [9, 10]. In this regard, MSU crystal-induced inflammation is more severe in SIRT1^+/−^ compared to WT mice [18]. Activation of SIRT1 suppresses MSU crystal-induced inflammatory responses [18, 19]. Since resident macrophages initiates and drives inflammation in response to MSU crystals [5–7] and that CD38 is responsible for NAD^+^ decline in inflammatory macrophages [14–16], we investigated the role of CD38 and NAD^+^ in macrophage responses to MSU crystals and in a model of acute gouty inflammation.

## Materials and methods

### Reagents

All chemicals were from Sigma-Aldrich (St. Louis, MO) unless otherwise indicated. Apigenin and 78c were obtained from MedChemExpress (Monmouth Junction, NJ). CD38 (# ab108403, RRID:AB_10890803) and acetylated SOD2 (K68) (#ab137037, RRID:AB_2784527) antibodies were from Abcam (Waltham, MA). NLRP3 (#15101, RRID:AB_2722591), caspase-1 (#24232, RRID:AB_2890194), SIRT3 (#5490, RRID:AB_10828246) and SOD2 (#13141, RRID:AB_2636921) antibodies were from Cell Signaling Technology, Inc (Danvers, MA). Cleaved caspase-1 (p10) antibody (#AG-20B-0044-C100, RRID:AB_2490253) from Adipogen Life Sciences (San Diego, CA).

### Preparing MSU crystals

MSU crystals were prepared under pyrogen-free conditions. In brief, 4 g uric acid pretreated for 2 h at 200 °C was dissolved in 775 ml of sterile deionized water and 25 ml sodium hydroxide (NaOH), which was then heated to 60 °C and adjusted to pH 7.5 with NaOH to let uric acid completely dissolved. The solution was left at room temperature overnight to allow formation of MSU crystals. The crystals were recovered by centrifugation, washed with 75% ethanol once followed by distilled water once, dried at 60 °C and resuspended in sterile PBS at 25 mg/ml. Crystal shape and birefringence were assessed by polarized light microscopy. MSU crystals were confirmed to be free of detectable endotoxin contamination (< 0.01 endotoxin Units/ml) by the limulus amebocyte lysate (LAL) assay using the Pierce™ Chromogenic Endotoxin Quant Kit (#A39552S, ThermoFisher, Waltham, MA).

### Frozen human whole blood and peripheral blood mononuclear cells (PBMCs)

The frozen human whole blood samples from healthy individuals were given by Dr. Jacob Karsh, University of Ottawa, Ottawa, Canada. The frozen human whole blood and PBMC pellet samples of gout patients from the UAB Rheumatology Arthritis Database and Repository (RADAR) cohort were provided by Dr. S.L. Bridges at University of Alabama, Birmingham, Alabama (UAB), USA.

All these samples were de-identified. The whole blood samples were obtained by collecting venous blood into BD Vacutainer Venous Blood Collection Tubes containing spray-dried K2 EDTA. The Ficoll-Hypaque density centrifugation method was used to isolate PBMCs from venous whole blood. All procedures were performed with institutionally peer reviewed, approved protocols.

### Mice

CD38KO (RRID: IMSR_JAX:003727) and WT mice (either male or female) on the C57BL6/J background were used for the study. All animal procedures were humanely performed with institutionally peer reviewed, approved protocols at VA San Diego.

### Subcutaneous air pouch model

The murine air pouch is a bursa-like space with the formation of a connective tissue cavity lined with cells which both structurally and functionally resembled synovial lining cells [20]. Injection of monosodium urate (MSU) crystals into the pouch elicits an acute inflammatory response like human gout [21]. Air pouches were generated on the back of 8–10-week-old mice by subcutaneous injection of 3 ml sterile-filtered air. Pouches were re-inflated on day 4 with an additional 3 ml filtered air. On day 7, MSU crystals (3 mg) in 1 ml of sterile, endotoxin-free PBS were injected into the pouch. Where indicated, apigenin (20 mg/kg/day) or nicotinamide riboside (NR) (400 mg/kg/day) were orally administered (gavage) to mice daily 3 days before injection of MSU crystals. Mice were euthanized 6 h after MSU crystal injection. For each mouse, 2 ml PBS containing 5 mM EDTA was injected into the pouch followed by gentle massage. Pouch fluid was then collected with syringes, and numbers of cells in pouch fluid were counted manually using a hemocytometer. After centrifugation of pouch fluid, supernatant was harvested and subjected to ELISA analysis of cytokines. The sample size of the air pouch model study was determined according to the design of 2 independent study groups (e.g., MSU vs. MSU + apigenin). N = 9 per group was calculated based on the mean difference of 0.5 (50%) between 2 groups with the standard deviation of 0.35, alpha (two sided) level of 0.05 and power of 80%.

### Histological and immunohistochemical (IHC) analysis of air pouch tissue

Air pouch tissues were dissected, fixed (in 10% formalin), embedded and sectioned for histological analysis by staining with Hematoxylin and Eosin Stain kit (#H-3502, Vector Lab, Newark, CA). Inflammation of pouch linings was evaluated semi-quantitatively based on hyperplasia/enlargement of lining layer and inflammatory cell infiltration with 0 = normal, 1 = mild, 2 = moderate, and 3 = severe changes. Pouch lining CD38 expression was examined by IHC analysis using a rabbit CD38 polyclonal antibody (#A13611, ABclonal, Woburn, MA).

### Isolation and culture of mouse bone marrow derived macrophages (BMDMs)

Briefly, bone marrow cells were isolated from the femurs and tibias of the mice by flushing the medullary cavity with PBS containing 2% fetal calf serum (FCS). After washing once in PBS, cells were cultured in RPMI containing 10% FBS, penicillin (100 U/ml), streptomycin (100 μg/ml) and 20% L929 conditioned media for 7 days to generate BMDMs. After priming with GM-CSF (20 ng/ml) for 24 h, cells were washed with PBS and subjected to experiments in RPMI containing 1% FBS.

### Quantitative RT-PCR

Total RNA was extracted using the RNeasy Mini kit (#74004) from Qiagen, (Germantown, MD) followed by reverse transcription to generate cDNA using the First Strand cDNA Synthesis kit (#11483188001, MilliporeSigma, Burlington, MA). The cDNAs were subjected to quantitative PCR analysis for expression of *CD38, NLRP3*, *Steap4*, *Olr1* and *Gpr176* using TaqMan Gene Expression Assay probe sets (ThermoFisher, Waltham, MA) for *CD38* (Mm01220906_m1), *NLRP3* (Mm00840904_m1), *Steap4* (Mm00475405_m1), *Ch25h* (Mm00515486_s1), *Olr1* (Mm00454582_m1), *Gpr176* Mm01277657_m1) and endogenous control *HPRT* (Mm03024075_m1). The data were analyzed using the ΔΔCT method.

### RNA sequencing (RNA-seq) and data analysis

After priming with GM-CSF (20 ng/ml) for 24 h, WT BMDMs in the presence or absence of apigenin and CD38KO BMDMs were stimulated with MSU crystals for 6 h. Cells were collected and subjected to RNA-seq analysis, performed by LC Sciences (Houston, TX). Kyoto Encyclopedia of Genes and Genomes (KEGG) was used for pathway enrichment analysis of differentially expressed genes (DEGs). STRING application-Protein Query was employed to generate a node network of functional enriched DEGs with defined molecular functions [Gene Ontology (GO)] for each cluster.

### Western blotting

Cells were lysed in RIPA buffer containing protease inhibitor cocktails (Roche, Mannheim, Germany). Cell lysates (10–15 μg) were separated by gradient 4–20% SDS-PAGE and transferred onto Immobilon^®^ PVDF membranes (MilliporeSigma, Burlington, MA), probed with primary followed by near-infrared secondary antibodies. The membranes were scanned on the LI-COR Odyssey imaging system (LI-COR Biotech, Lincolin, NB).

### Measurement of NAD^+^ and NADH and mitochondrial superoxide

NAD^+^ content in de-identified frozen human whole blood samples or mouse whole blood samples collected via cardio puncture immediately after euthanasia were measured by liquid chromatography–mass spectrometry (LC–MS) at the mass spectrometry core, University of South Alabama (detailed description in the supplemental materials). Macrophage intracellular NAD^+^ and NADH (the reduced form of NAD^+^) content were measured using a NAD^+^/NADH quantification colorimetric kit (#K337, BioVision, Milpitas, CA). NAD^+^ to NADH ratio was determined. Mitochondrial superoxide generation was assessed by MitoSOX Red (#M36008, ThermoFisher) staining.

### Cytokine analyses

IL-1β and CXCL1 (KC) were measured using DuoSet ELISA (#DY401 and #DY453, R&D Systems, Minneapolis, MN).

### Statistical analyses

GraphPad PRISM 9 (San Diego, CA) was used for statistical analyses. All data were subjected to the normality test. For normally distributed data, unpaired student t-test (comparing 2 groups, 2-sided, alpha = 0.05) or two-way *ANOVA* with Tukey multiple comparisons test (comparing 2 ≥ groups with 2 independent variables, alpha = 0.05) were performed. The data were expressed as mean ± SD or mean ± SEM. *P* < 0.05 was considered statistically significant.

## Results

### ***Decreased blood NAD***^+^***levels and increased CD38 expression in PBMCs of gout patients***

**Fig. 1:**
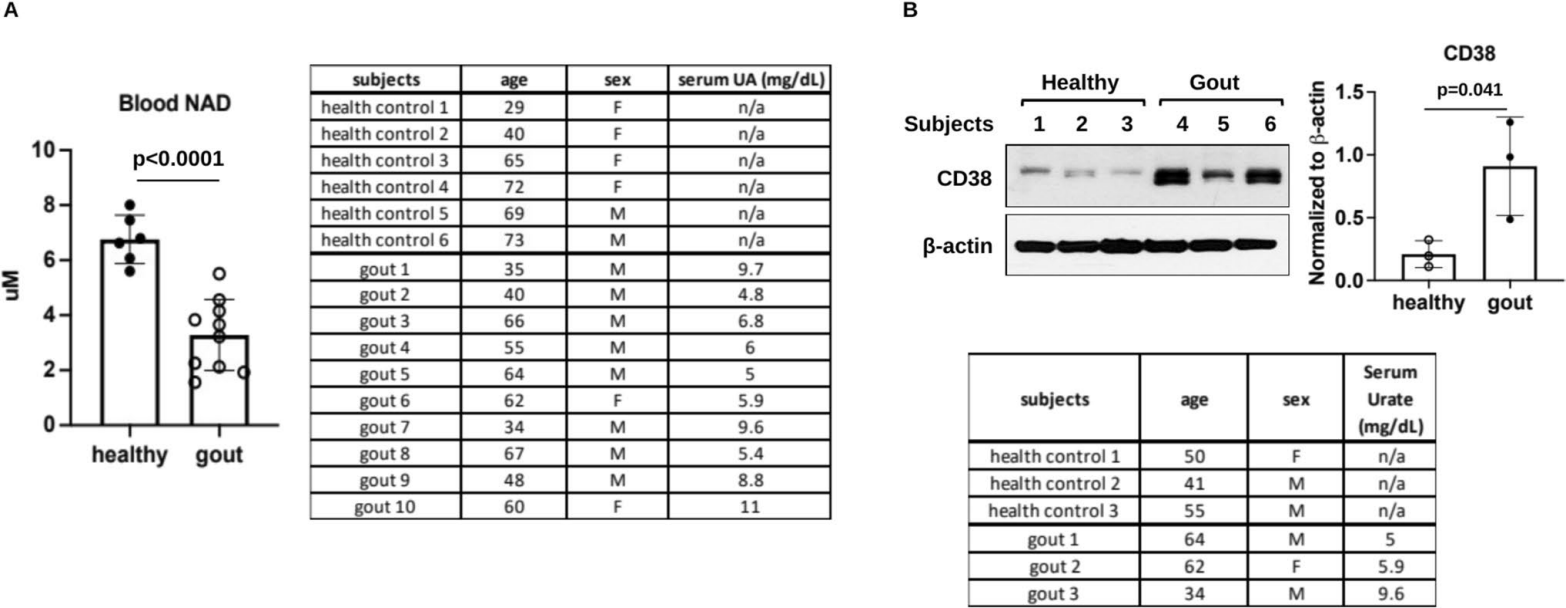
Decreased blood NAD^+^ levels and increased PBMC CD38 expression in gout patients. Whole blood samples from 6 healthy controls and 10 gout patients with flare at the time of blood draw were subjected LC-MS analysis of NAD^+^ content (**A**). PBMC cell pellets from 3 healthy controls and 3 gout patients were used for Western blot analysis of CD38 expression followed by semi-quantitative densitometry analysis (**B**). The subject’s age, sex and serum uric acid levels were listed in the tables in A and B. Data in A were expressed as mean ± SD. Statistical analysis was performed using the Student t-test

In limited, seminal studies, LC–MS analysis of whole blood samples from 6 healthy controls and 10 gout patients with acute gout flare revealed that the mean NAD^+^ levels were significantly decreased in gout patients (3.28 ± 1.29 µM) compared to healthy controls (6.76 ± 0.88 µM) (Fig. 1A), suggesting systemic NAD^+^ decline in gout patients. Western blot analysis of cell lysates of PBMCs from 3 healthy controls and 3 gout patients showed increased CD38 expression in gout patients compared to healthy controls (Fig. 1B). This prompted us to study the role of CD38 in MSU crystal-induced inflammatory responses in macrophages in vitro and in the mouse air pouch gout model in vivo.

### Induction of CD38 expression by MSU crystals and associated effects on NAD^+^/NADH and macrophage cytokine expression

**Fig. 2:**
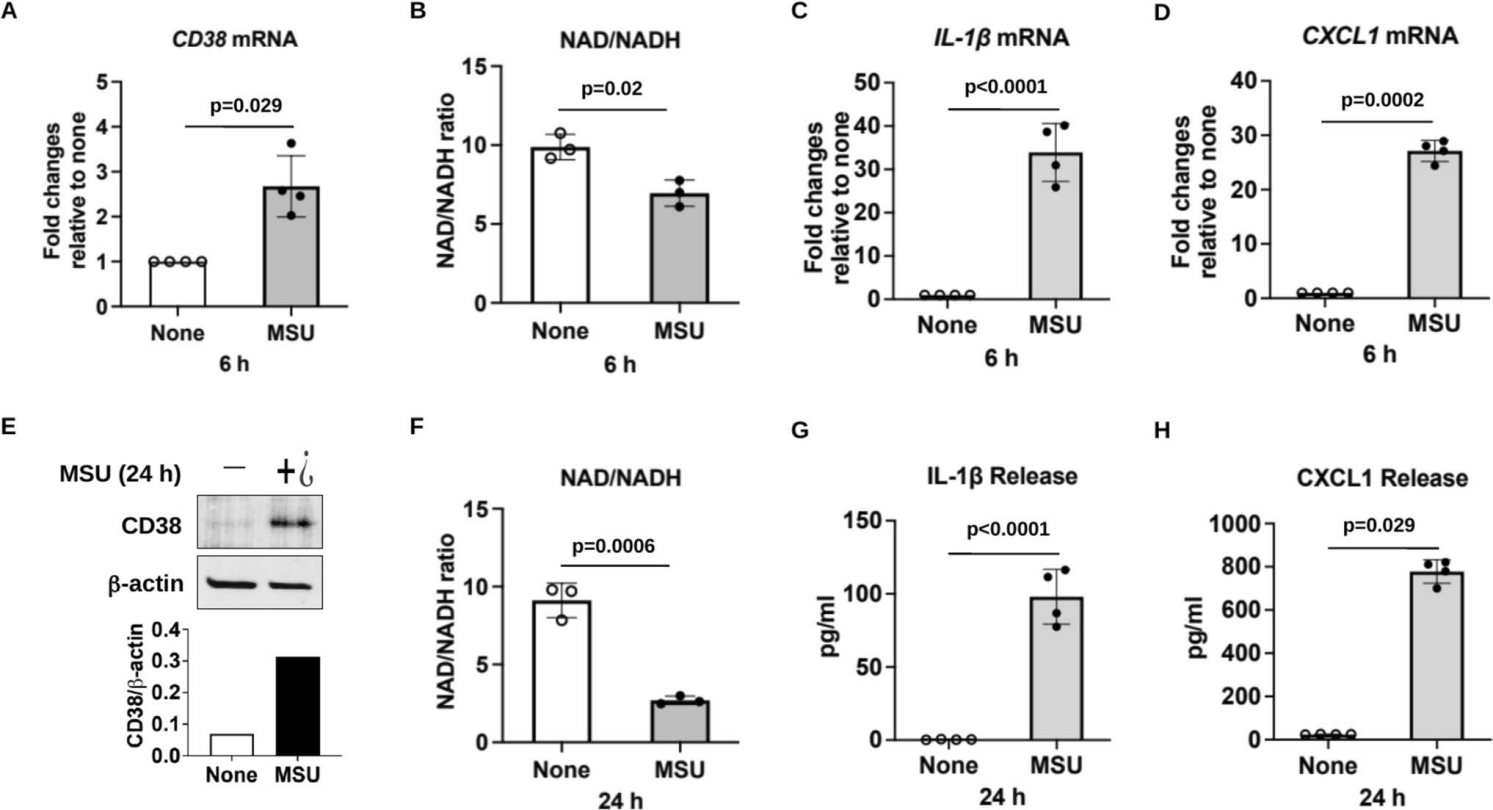
Upregulation of CD38 expression was correlated with decreased intracellular NAD^+^/NADH and increased cytokine production in MSU crystal-treated mouse BMDMs in vitro*.* BMDMs were stimulated with MSU crystals (0.2 mg/ml) for 6 and 24 hours, and then subjected to qRT-PCR for mRNA expression of CD38, IL-1β and CXCL1 (**A**, **C**, **D**) and Western blot for CD38 protein expression followed by semi-quantitative densitometry analysis (**E**), respectively. Release of IL-1β and CXCL1 was assessed by ELSA (**G**, **H**). Cellular NAD^+^ and NADH were measured and expressed as NAD^+^/NADH representing the cell redox state (**B** and **F**). Data were generated with 3–4 biological replicates and expressed as mean ± SD. Statistical analyses for were performed using the nonparametric Mann–Whitney test for **A** and **H** and the Student *t*-test for **B**–**D**, **F** and **G**

After stimulation of mouse BMDMs with MSU crystals for 6 and 24 h, CD38 was upregulated at mRNA and protein levels, respectively (Fig. 2A–E). Intracellular levels of NAD^+^/NADH, a key measure of cellular redox state reflecting both total metabolic function and the cellular health status [22, 23], were significantly reduced at both time points (Fig. 2B–F), associated with induction of IL-1β and the chemokine CXCL1 at both mRNA (Fig. 2C, D) and protein levels (Fig. 2G, H). These results suggested an association of decreased intracellular NAD^+^/NADH with the inflammatory responses to MSU crystals in macrophages.

### Effects of CD38 expression on macrophage responses to MSU crystals

**Fig. 3:**
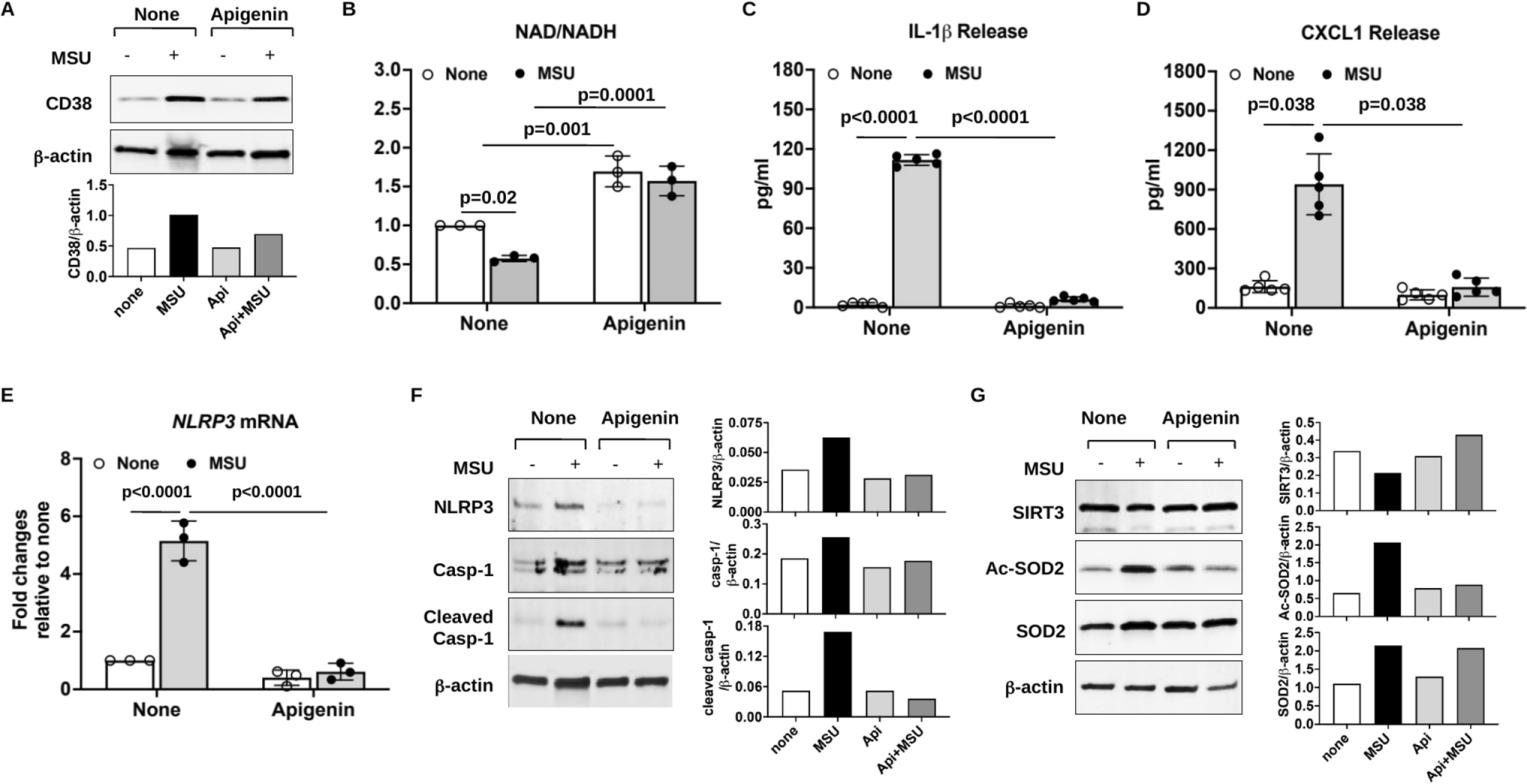
Inhibition of CD38 expression by apigenin elevated intracellular NAD^+^/NADH levels and exhibited anti-inflammatory effects in MSU crystal-treated mouse BMDMs in vitro. BMDMs were stimulated with MSU crystals (0.2 mg/ml) in the presence or absence of apigenin (25 µM) for 6 hours (**E**) and 24 hours (**A**–**D**, **F**, **G**). Expression of CD38 (**A**) and NLRP3, pro-caspase-1 and cleaved caspase-1, SIRT3, acetylated SOD2, and SOD2 (**F** and **G**) was examined by Western blot followed by semi-quantitative densitometry analysis. NLRP3 mRNA expression was assessed by qRT-PCR analysis of BMDMs received 6 hours treatment (**E**). NAD^+^/NADH was determined after measuring cellular NAD^+^ and NADH (**B**). Release of IL-1β and CXCL1 was quantified by ELISA (**C** and **D**). Data in **A**, **F** and **G** were representative of 3 independent experiments. Data in B–E were generated with 3–5 biological replicates and expressed as mean ± SD. Statistical analysis was performed using the Two-way *ANOVA* with Tukey multiple comparison test for **B**, **C**, **E** and the nonparametric Kruskal–Wallis test for **D**

We assessed the effects of inhibition of CD38 by apigenin, a natural flavonoid CD38 inhibitor [24], in macrophages stimulated with MSU crystals in vitro. As shown in Fig. 3, apigenin inhibited induction of CD38 expression (**A**), increased basal and reverted MSU crystal-induced reduction of cellular NAD^+^/NADH (**B**). This was mainly due to ability of apigenin to reduce basal levels of NADH and prevent an increase in NADH levels induced by MSU crystals (Supplemental Table 1). The results also aligned with attenuation of IL-1β and CXCL1 release (Fig. 3C, D) induced by MSU crystals. We then tested specificity of the effects of CD38 inhibition, using either a more selective CD38 pharmacological inhibitor 78c or CD38KO BMDMs. IL-1β and CXCL1 induction by MSU crystals were blunted in BMDMs that either received 78c treatment or were genetically deficient in CD38 (Supplemental Figure S1).

Next, we determined the effect of apigenin on NLRP3 inflammasome activation in response to MSU crystals in macrophages. MSU crystals induced upregulation of NLRP3 expression at both mRNA and protein levels, which were diminished by apigenin (Fig. 3E, F). In addition, MSU crystal-induced expression of cleaved caspase-1 was attenuated by apigenin (Fig. 3F). Mitochondrial reactive oxygen species (ROS) centrally mediate NLRP3 inflammasome activation [25], and mitochondrially located SIRT3 plays an important role in regulating oxidative stress by deacetylation of substrates involved in ROS production [26]. Hence, we examined levels of SIRT3 and acetylation of superoxide dismutase (SOD2), an antioxidant enzyme located in mitochondria, as well as mitochondrial superoxide generation. Decreased SIRT3 expression and increased acetylation of SOD2 (Fig. 3G), associated with increased MitoSOX Red staining (Supplemental Figure S2) were observed in BMDMs stimulated with MSU crystals, which were reversed by apigenin. These results suggested that apigenin inhibited NLRP3 inflammasome activation at least partly by alleviating mitochondrial oxidative stress via SIRT3-SOD2 signalling.

### Apigenin suppressed MSU crystal-induced inflammation in vivo

**Fig. 4:**
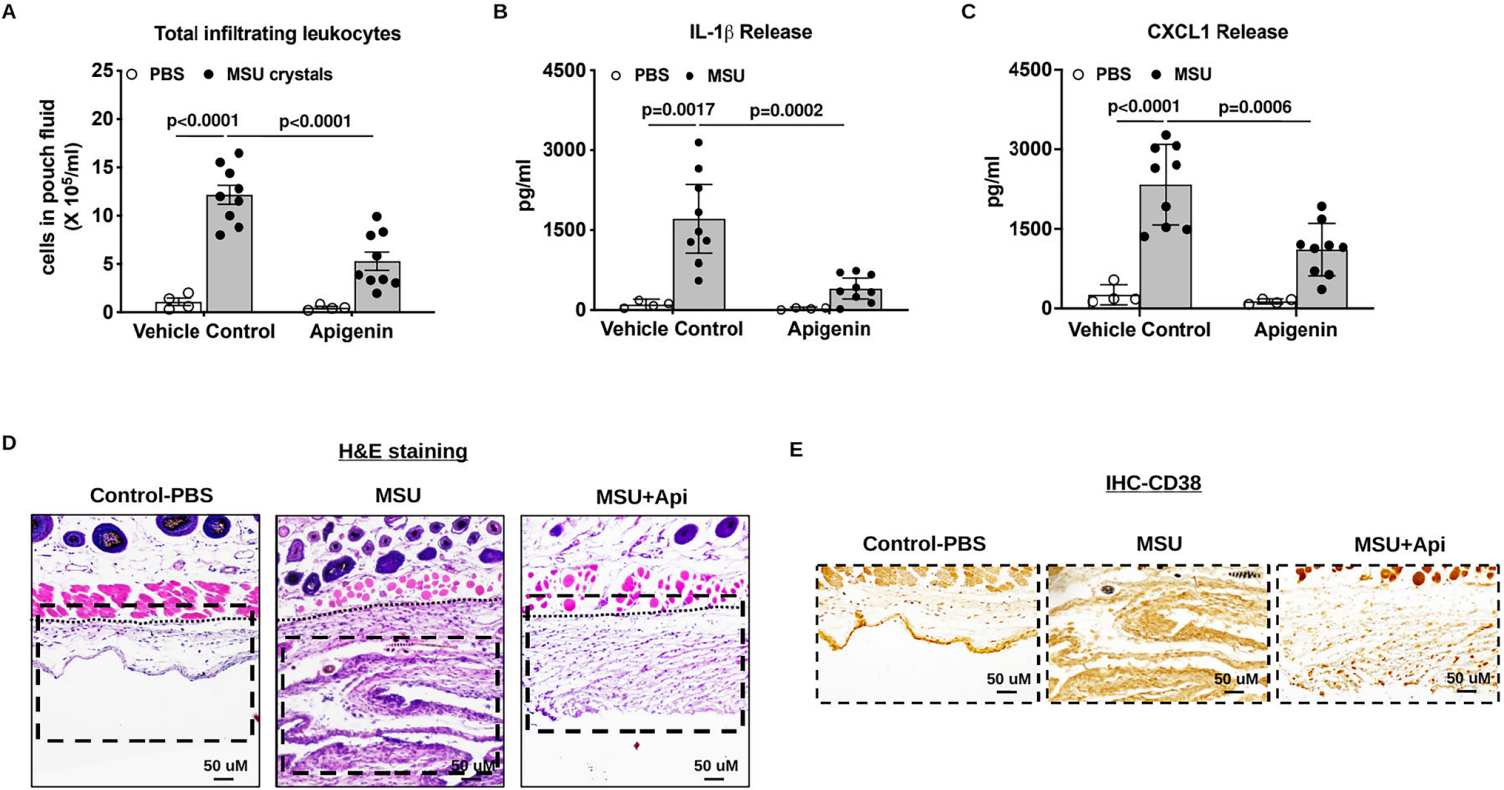
Apigenin ameliorated MSU crystal-induced inflammation in the mouse air pouch model in vivo. Air pouches were created in C57BL/6 mice, and apigenin was orally administered to mice. Acute inflammatory responses to MSU crystals were determined by measuring the numbers of infiltrating cells and production of IL-1β and CXCL1 in the air pouch exudate 6 hours post-injection (**A**, **B** and **C**), and by H&E staining (**D**) and CD38 IHC analysis (**E**) of air pouch tissues. The inflammation scores of pouch lining (below the dotted line) based on H&E staining for MSU and MSU+apigenin groups were 3 and 1 respectively, compared to 0 in the PBS control group. The score for the apigenin only group was also 0 (image was not shown). Data in **A**–**C** were generated with 9 biological replicates (mice) per group and expressed as mean with 95% CI. Statistical analysis was performed using Two-way *ANOVA* with Tukey multiple comparison test for **A**, **C** and the nonparametric Kruskal–Wallis test for **B**

The murine subcutaneous air pouch model was used to determine the effect of apigenin in MSU crystal-induced inflammation in vivo. MSU crystal-stimulated mice that received apigenin treatment exhibited significantly lower numbers of leukocytes infiltrated in the pouch fluid (Fig. 4A). In addition, MSU crystal-induced IL-1β and CXCL1 release in the pouch were significantly inhibited by apigenin (Fig. 4B, C). H&E staining of the air pouch tissue sections of these mice revealed that the pouch lining thickness and cell infiltration were evidently reduced with the inflammation score of 1 (mild), compared to that of 3 (severe) in mice stimulated with MSU crystals only (Fig. 4D). Reduced CD38 expression analysed by IHC was also observed in MSU crystal-stimulated mice that received apigenin treatment (Fig. 4E). Similarly, MSU crystal-induced leukocyte infiltration and release of IL-1β and CXCL1 were attenuated in CD38KO compared to WT mice (Supplemental Figure. S3).

### Effects of CD38 pharmacologic inhibition or genetic knockout on gene expression in MSU crystal-stimulated macrophages

**Fig. 5:**
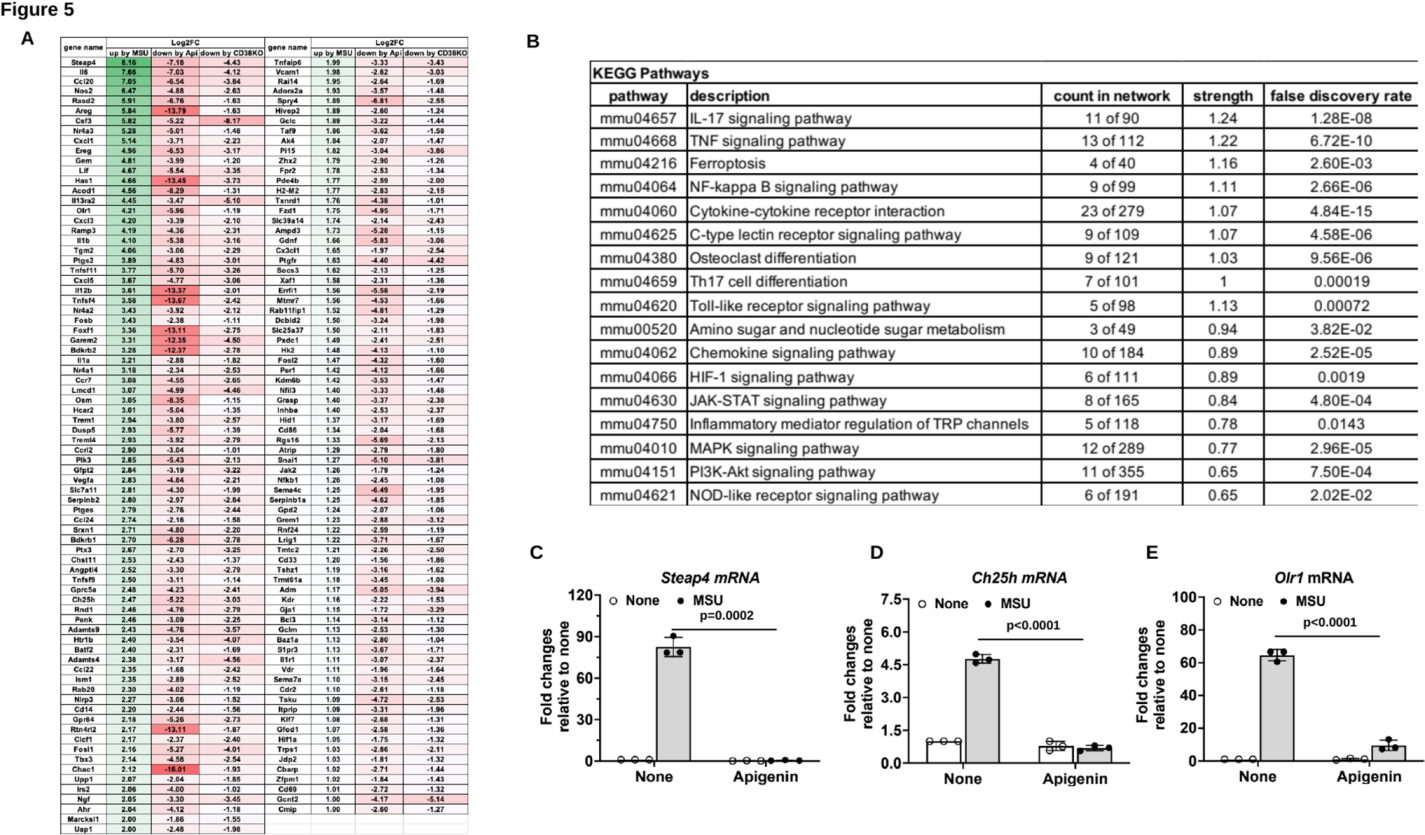
Analysis of DEGs that were upregulated by MSU crystals but downregulated by both apigenin and CD38 knockout in mouse BMDMs. 154 DEGs that were identified to be upregulated in WT BMDMs+MSU but downregulated in WT BMDMs+MSU+apigenin and CD38KO BMDMs+MSU (**A**) were subjected to KEGG pathway enrichment analysis (**B**). Expression of selected DEGs *Steap4*, *Ch25h* and *Olr1* was validated by qRT-PCR analysis of mouse BMDMs stimulated with MSU crystals in the presence or absence of apigenin for 6 hours (**C**–**E**). Data in **C**–**E** were expressed as mean ± SD. Statistical analysis was performed using the nonparametric Kruskal-Wallis test for **C** and Two-way *ANOVA* with Tukey multiple comparison test for **D** and **E**

We performed RNA-seq to identify differentially expressed genes (DEGs) and effects of CD38 deficiency in macrophages stimulated with MSU crystals. Of the 507 genes upregulated (log_2_FC ≥ 1) by MSU crystals in WT BMDMs (WT + MSU vs WT non-treated), 401 and 207 were downregulated (log_2_FC ≤ − 1) by apigenin in WT (WT + MSU + Api vs WT + MSU) and CD38KO (CD38KO + MSU vs WT + MSU) BMDMs, respectively, of which 154 genes were common (Fig. 5A, mean log_2_FC values of DEGs, and supplemental Figure S4, heatmap of DEGs with biological replicates). Many of the shared changes were in inflammatory genes.

STRING enrichment analysis of the 154 DEGs displayed 3 major clusters (Supplemental Figure S5A) with molecular function (GO) in chemokine and cytokine activities, chemokine and cytokine receptor binding, nuclear receptor activity, transcription coactivator or cofactor binding, growth factor activity and growth factor receptor binding (cluster 1), Bradykinin receptor activity (cluster 2) and glutamate-cysteine ligase activity (cluster 3) (Supplemental Figure S5B). KEGG pathway enrichment analysis of these 154 DEGs revealed several signalling pathways implicated in inflammation. These included IL-17, TNF, chemokine, NF-κB, C-type lectin receptor, Toll-like receptor, JAK/STAT, MAPK, PI3K/Akt, and NOD-like receptor signalling pathways, as well as ferroptosis and osteoclast differentiation (Fig. 5B).

Six-transmembrane epithelial antigen of prostate 4 (*Steap4*), a metalloreductase involved in iron homeostasis, was found to be the top DEG upregulated in WT BMDMs (log_2_FC = 8.2) but was downregulated in CD38KO (log_2_FC = − 4.4) and apigenin-treated WT (log_2_FC = − 7.2) BMDMs stimulated with MSU crystals. Notably, some genes involved in cholesterol and lipid metabolism and signalling such as cholesterol 25-hydroxylase (*Ch25h*) and oxLDL receptor 1 (*Olr1*), also known as lectin-like oxidized low-density lipoprotein receptor-1 (*Lox-1)*, were upregulated in MSU crystal-stimulated WT BMDMs, but were downregulated in MSU crystal-stimulated CD38KO BMDMs and WT BMDMs in the presence of apigenin. Gene expression changes of *Steap4, Ch25h* and* Olr1* were validated by qRT-PCR analysis of BMDMs stimulated with MSU crystals in the presence of apigenin (Fig. 5C–E).

**Fig. 6:**
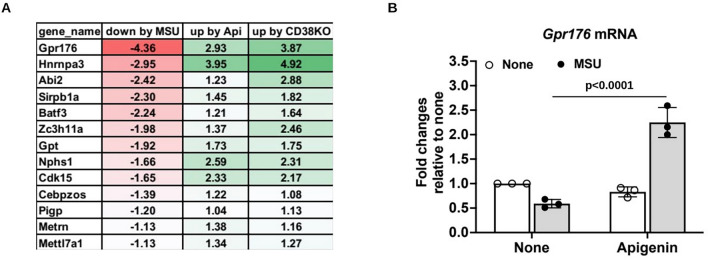
Analysis of DEGs that were downregulated by MSU crystals but upregulated by both apigenin and CD38 knockout in mouse BMDMs. 13 DEGs were identified to be downregulated in WT BMDMs+MSU but upregulated in WT BMDMs+MSU+apigenin and CD38KO BMDMs+MSU (**A**). Expression of *Gpr176* was validated by qRT-PCR analysis of mouse BMDMs stimulated with MSU crystals in the presence or absence of apigenin for 6 hours (**B**). Data in **B** were expressed as mean ± SD. Statistical analysis was performed using Two-way *ANOVA* with Tukey multiple comparison test

Of the 527 downregulated (log_2_FC ≤− 1) genes by MSU crystals in WT BMDMs, 64 and 48 genes were upregulated by apigenin in WT BMDMs and CD38KO BMDMs, respectively, of which 13 genes were common shown in Fig. 6A (mean log_2_FC values of DEGs) and Supplemental Fig. 4B (heatmap of DEGs with biological replicates). *Gpr176*, a Gz-linked orphan G protein-coupled receptor (GPCR), was the top DEG downregulated by MSU crystals (log_2_FC < −4) but was upregulated by apigenin or CD38 deficiency. This result was validated by qRT-PCR in BMDMs stimulated with MSU crystals in the presence of apigenin (Fig. 6B).

### Nicotinamide Riboside (NR) supplementation attenuated MSU crystal-induced inflammatory responses

**Fig. 7:**
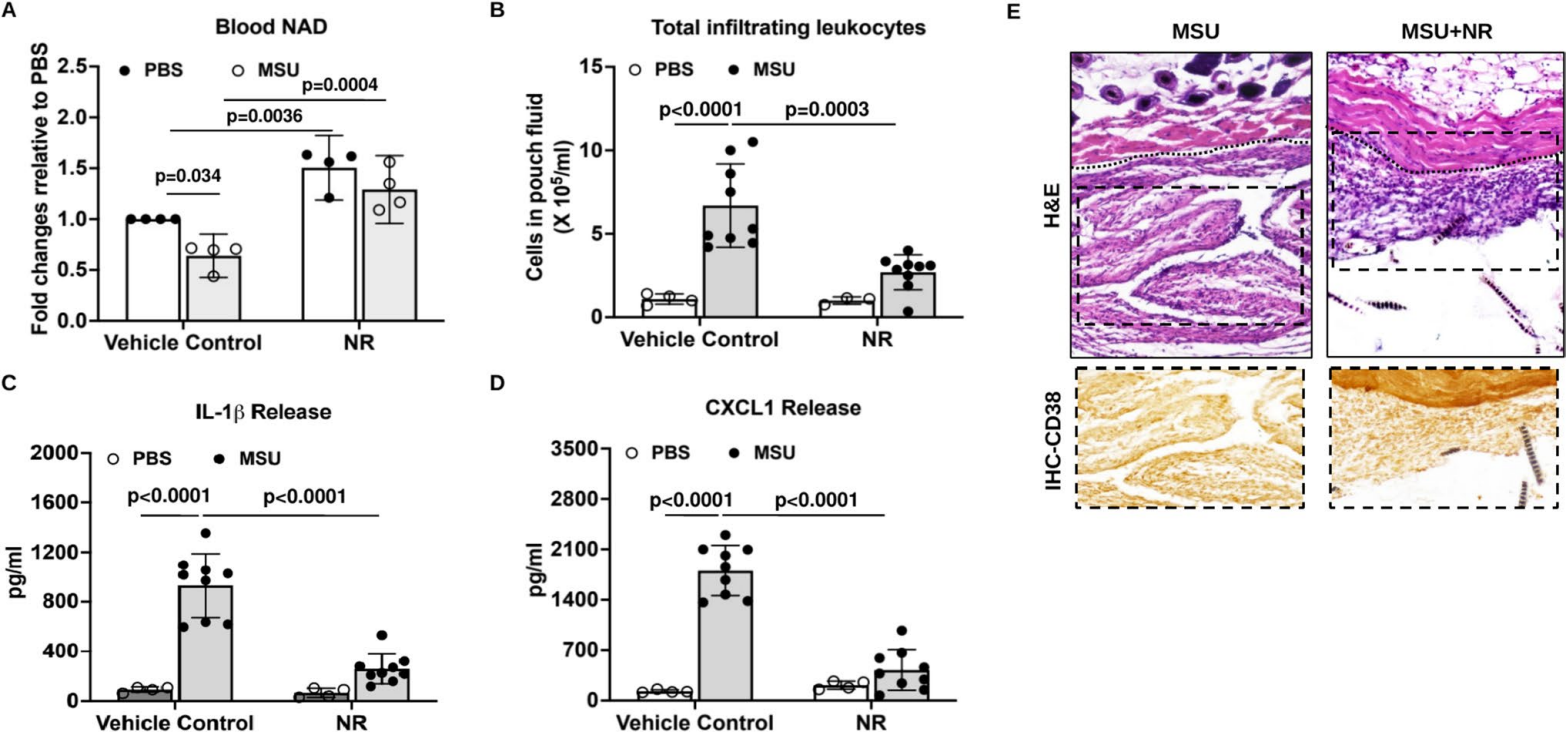
Nicotinamide Riboside (NR) supplementation attenuated MSU crystal-induced inflammatory responses. Air pouches were created, and NR was orally administered to mice. The whole blood NAD^+^ levels were measured by LC-MS and expressed as fold changes relative to the PBS control (**A**). The numbers of infiltrating cells (**B**) and production of IL-1β and CXCL1 (**C**, **D**) in the air pouch exudate 6 hours post-injection were assessed. H&E staining and CD38 IHC analysis (**E**) of air pouch tissue sections were performed. The inflammation scores of pouch lining (below the dotted line) for MSU and MSU+NR groups were 3 and 1, respectively. Data in A were generated with 4 biological replicates (mice) per group and expressed as mean ± SD. Data in **B**–**D** were generated with 9 biological replicates (mice) per group and expressed as mean with 95% CI. Statistical analysis was performed using Two-way *ANOVA* with Tukey multiple comparison test for **A**–**D**

Oral administration of NR, a bioavailable NAD^+^ precursor that has boosted NAD^+^ levels and exerted anti-inflammatory effects in clinical studies [27–29]. In our in vivo air pouch model study, mice received oral supplementation of NR showed elevated basal and reversed MSU crystal-induced decrease in systemic NAD^+^ levels (Fig. 7A). In addition, MSU crystal-induced leukocyte infiltration (Fig. 7B) and release of IL-1β and CXCL1 in the pouch fluid were greatly inhibited by NR (Fig. 7C, D). For MSU crystal-stimulated mice received oral supplementation, the pouch lining thickness and cell infiltration were evidently reduced, correlated with less CD38 expression (Fig. 7E). Moreover, NR significantly inhibited MSU crystal-induced gene expression of *CD38*, *Steap4*, *Ch25h* and *Olr1* and reversed MSU crystal-induced reduction of *Gpr176* in macrophages in vitro (Supplemental Figure S6).

## Discussion

In this study, we confirmed induction of CD38 by MSU crystals, and elucidated the association with significantly reduced intracellular levels of NAD^+^/NADH, in mouse BMDMs in vitro. These effects were reversed by apigenin, a natural flavonoid with ability to block CD38 NADase activity [24]. Importantly, inhibition of CD38 either by two pharmacological inhibitors (apigenin or the more specific compound 78c) or CD38 genetic knockout suppressed MSU crystal-induced release of IL-1β and CXCL1 in BMDMs. Moreover, apigenin-treated or CD38KO mice had blunted inflammatory responses to MSU crystals in vivo.

CD38 induction by MSU crystals was likely mediated by activation of transcription factors NF-κB and STAT, whose binding sites are in the CD38 gene promoter. Both NF-κB and JAK/STAT signalling pathways were highlighted in our KEGG pathway enrichment analysis of genes upregulated by MSU crystals but downregulated by both apigenin and CD38 deficiency. Inhibition of MSU crystal-induced NRLP3 gene expression by apigenin may also act on by inactivation of NF-κB signalling since NF-κB-dependent signals regulate NLRP3 expression. Shim et al. showed that intracellular NAD^+^ decline can provide a non-transcriptional priming signal for NLRP3 inflammasome activation by causing mitochondrial retrograde transport [12]. A lower concentration of intracellular NAD^+^ can lead to increased acetylation of α-tubulin via inhibition of NAD^+^-dependent SIRT2 activity [30].

Regulation and maintaining a proper balance of the NAD^+^/NADH ratio is critical for normal cell function and viability. A decrease in the cellular NAD^+^/NADH ratio, reduced NAD^+^ levels and increased NADH levels have all been observed in aging [31]. NAD^+^/NADH redox imbalance can lead to oxidative stress [32]. Reduced NAD^+^/NADH ratio has been strongly implicated in mitochondrial dysfunction due to suppression of SIRT3 activity [33]. Several studies have shown that mitochondrial SIRT3-mediated activation of SOD2 signalling inactivates NLRP3 inflammasome activation [34–37]. We observed decrease in NAD^+^/NADH ratio and SIRT3 expression and increase in acetylation of SOD2 and mitochondrial superoxide generation in macrophages stimulated with MSU crystals. These effects were limited by apigenin treatment. Hence, apigenin may act on preserving the NAD^+^/NADH balance and activating SIRT3-SOD2 signalling to suppress MSU crystal-induced NLRP3 inflammasome activation by inhibiting generation of mitochondrial ROS.

CD38 consumes NAD^+^ to form NAM, ADPR and to a less extent cADPR [9, 10]. Both ADPR and cADPR can act as second messengers controlling cell functions through calcium (Ca^2+^) mobilization [9, 10]. Ca^2+^ mobilization also plays a critical role in NLRP3 inflammasome activation [38]. CD38 regulates NLRP3 inflammasome activation through cADPR-mediated Ca^2+^ release in vascular smooth muscle cells in diabetic mice [39]. Whether CD38 regulates NLRP3 inflammasome activation by MSU crystals through ADPR or cADPR-mediated Ca^2+^ mobilization in macrophages remains to be determined.

In this study, more genes upregulated or downregulated by MSU crystals in WT BMDMs were reversed by apigenin treatment (401 or 207 genes, respectively) than by CD38 genetic knockout (64 or 48 genes, respectively). Because apigenin is a selective but not specific CD38 inhibitor, to focus on CD38-targeting effects of apigenin, we performed subsequent analyses with those MSU crystal-induced DEGs (upregulated and downregulated) that were commonly reversed by apigenin and by CD38 knockout. Inhibition of CD38 suppressed several other inflammatory signalling pathways activated by MSU crystals, especially pathways for IL-17 signalling and Th17 cell differentiation, which were also enriched in our previous genome-wide DNA methylation analyses of PBMCs of gout patients [40], chemokine and cytokine receptor binding and activity, and nuclear receptor activity.

*Steap4* was identified as the DEG most robustly induced by MSU crystals that was blunted by both apigenin and CD38 knockout in macrophages. STEAP4 is a metalloreductase that reduces both Fe^3+^ to Fe^2+^ and Cu^2+^ to Cu^+^, which are prerequisites for the transport of these metals into cells [41]. Increased expression of STEAP4 increases iron and/or copper import into cells [41]. STEAP4 is linked to inflammation and innate immune response, and its expression is induced by inflammatory cytokines including IL-1β, TNFα, and IL-6 [41]. Copper levels are elevated in inflamed tissues. Increased STEAP4 enhances cellular copper uptake, activating E3-ligase activity of XIAP, resulting in sustained NF-κB activation [42]. Overexpression of STEAP4 can cause iron accumulation in mitochondria leading to enhanced ROS production and mitochondrial dysfunction, which can further amplify inflammation [43].

Notably, ferroptosis signalling pathway was identified in KEGG pathway enrichment analysis of 154 genes upregulated by MSU crystals but downregulated by apigenin and CD38 knockout. Ferroptosis is an iron-dependent cell death pathway with unique characteristics including lipid peroxide accumulation, mitochondrial cristae loss, and mitochondrial membrane rupture and condensation [44]. Ferroptosis is triggered by accumulation of iron and lipid peroxidation and is linked to NLRP3 inflammasome activation [44]. Given the ability of MSU crystals to robustly induce *Steap4* expression and promote macrophage mitochondrial cristae loss [45], it is conceivable that ferroptosis via mitochondrial iron accumulation is involved in MSU crystal-induced NLRP3 inflammasome activation.

STEAP4 also plays a critical role in osteoclastogenesis [46]. Enhanced monocyte/macrophage differentiation to osteoclasts, and increased osteoclastogenesis at the tophus-bone interface are believed to contribute to bone erosion in gouty arthritis [47]. Notably, osteoclast differentiation was one of enriched BMDM signalling pathways upregulated by MSU crystals but downregulated by apigenin and CD38 genetic knockout in the KEGG pathway analysis.

This study also revealed that apigenin and CD38 genetic knockout suppressed induction by MSU crystals of *Ch25h* and *Olr1*, genes involved in cholesterol and fatty acid metabolism and signalling in macrophages. *Ch25h* is a cholesterol 25-hydroxylase that converts cholesterol to 25-hydroxycholesterol (25-HC), an oxysterol acting as an innate immune mediator that can amplify inflammatory responses in macrophages [48]. OLR-1 is a transmembrane glycoprotein that binds a broad spectrum of structurally distinct ligands such as oxLDL, phosphatidylserine (PS), apoptotic bodies, advanced glycation end-products (AGEs), and Hsp60 [49, 50]. Following uptake of its ligand, OLR-1 induces inflammatory signalling pathways leading to production of ROS, secretion of inflammatory cytokines and induction of apoptosis [49, 50]. These data implicate that MSU crystal-induced inflammatory responses could be mediated through impaired iron homeostasis and dysregulated cholesterol and lipid metabolism and signalling in macrophages, all targetable by inhibition of CD38.

*Gpr176*, the DEG most robustly downregulated by MSU crystals in macrophages, was conversely upregulated by both apigenin and CD38 knockout. GPR176 is an orphan GPCR involved in normal circadian rhythm behaviour [51]. Studies have shown that expression of GPR176 is enriched in the suprachiasmatic nucleus (SCN), the brain's circadian pacemaker, and that GRP176 governs daily rhythms in behaviour and physiology [51]. Expression of GPR176 in the SCN is under the control of the core clock components *Cry1* and *Cry2* [51]. Circadian disruption can lead to dysregulation of immune responses and inflammation which can further interfere with circadian rhythms [52]. Gout flares, disproportionately frequent at night, appear significantly influenced by circadian rhythm, which regulates NLRP3 inflammasome activation, and phagocyte behaviour, and is subject to altered epigenomic modulation in gout patients [40, 53]. Hence, downregulation of GRP176 by MSU crystals, by altering circadian rhythm behaviour in macrophages, has the potential capacity to modulate inflammatory responses to MSU crystals.

Our demonstration of the capacity of oral supplementation of the NAD^+^ precursor NR to elevate systemic NAD^+^ levels and attenuate inflammatory responses to MSU crystals was noteworthy. Clinical trials have shown that oral supplementation of NR is well-tolerated, has boosted human NAD^+^ metabolism in a dose-dependent manner [54, 55], and exerts anti-inflammatory effects [27–29]. Therefore, translational strategies to boost NAD^+^ levels using oral NR supplementation warrant further clinical investigations for preventing and limiting gout flares.

In conclusion, CD38 deficiency through either pharmacologic inhibition or genetic knockout attenuated inflammatory responses to MSU crystals in macrophages in vitro and in the air pouch model in vivo. CD38 inhibition acts by preserving intracellular NAD^+^/NADH balance, which is associated with NAD^+^-dependent sirtuin signalling. Seminal studies suggested that gout patients may have lower systemic NAD^+^ levels and increased PBMC CD38 expression compared to healthy controls. Larger-scale analyses would be of interest but were beyond the scope of this study. Gout incidence and prevalence increase with age. In this context, CD38 expression and activity are increased during aging associated with NAD^+^ decline [9, 10]. Thus, inhibition of CD38 is identified as a novel candidate druggable approach to prevent and treat gouty inflammation, by correcting an aging-associated change.

### Supplementary Information

Below is the link to the electronic supplementary material.

Supplementary file1 (PDF 1779 KB)

## Data Availability

The data supporting the findings of this study are available within the article and its supplementary information files or from the corresponding author upon reasonable request. RNA-seq data is available in the Gene Expression Omnibus (GEO) database #GSE214587.
