# Supplementary material for: The NADase CD38 is a central regulator in gouty inflammation and a novel druggable therapeutic target
Source: Inflamm Res. 2024 Mar 16;73(5):739–51. doi: 10.1007/s00011-024-01863-y (PMC11058052; doi:10.1007/s00011-024-01863-y)
Supplement: Supplementary file 1 — Supplementary file1 (PDF 1779 KB) [file 11_2024_1863_MOESM1_ESM.pdf]

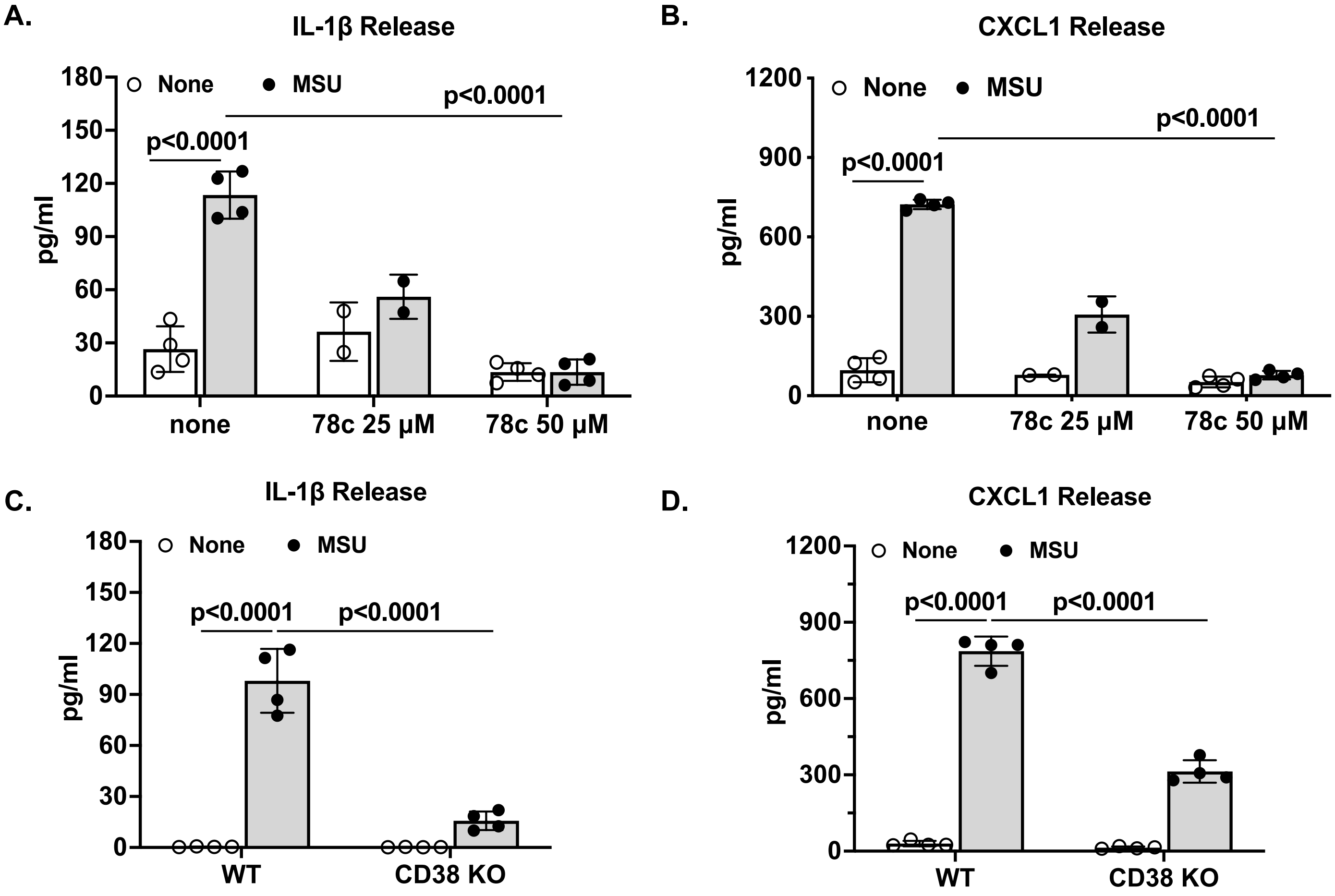

Supplemental Figure S1. Inhibition of MSU crystal-induced release of IL-1β and CXCL1 by 78c and CD38 deficiency in BMDMs *in vitro*. WT BMDMs in the presence of absence of CD38-specific inhibitor 78c (25 and 50 uM) and CD38KO BMDMs were stimulated with MSU crystals (0.2 mg/ml) for 24 hours. The conditioned media was used to quantify IL-1β and CXCL1 by ELISA analysis. Data were generated with 2-4 biological replicates and expressed as mean ±SD. Statistical analysis in A-D was performed using Two-way *ANOVA* with Tukey multiple comparison test. In A and B, data from 78c 25 uM group were not included in the statistical analysis.

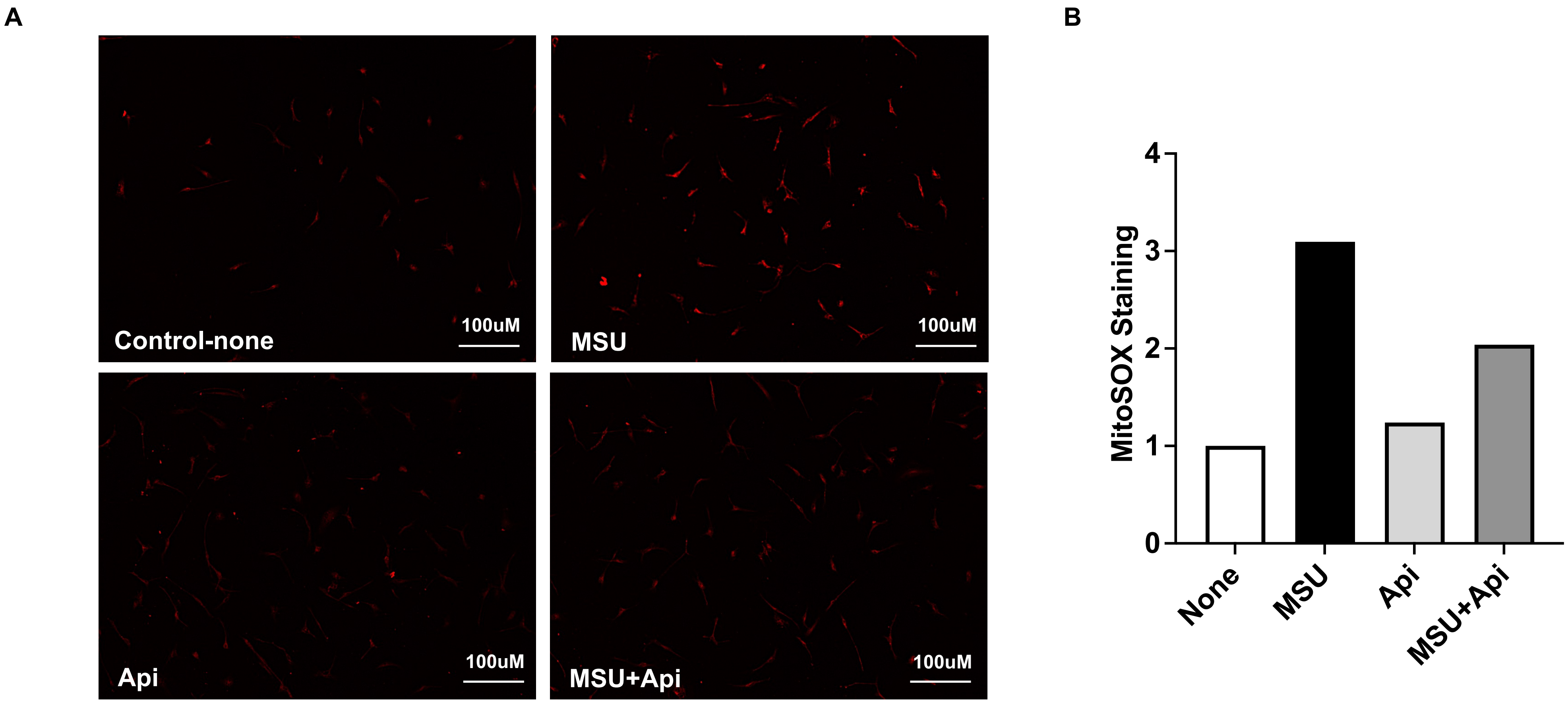

Supplemental Figure S2. Inhibition of MSU crystal-induced mitochondrial superoxide generation by apigenin in BMDMs in vitro. Mouse BMDMs were stimulated with MSU crystals (0.2 mg/ml) in the presence or absence of apigenin (25 uM) for 1 hour. MitoSOX Red staining (indicator of mitochondrial superoxide) were performed by incubating the cells with MitoSOX Red reagent (Theromo Scientific) reagent (1 uM), which was visualized in fluorescence Microscopy (A). All images underwent an 8-bit conversion, followed by contrast enhancement and threshold application. Subsequently, the intensity was analyzed using Image J and presented in the graph (B). Stronger red fluorescence signals were observed in cells treated with MSU crystals, compared to non-treated control. These were not seen when apigenin was present in the cells (MSU+Api), suggesting that apigenin can inhibit MSU crystal-induced mitochondrial superoxide generation.

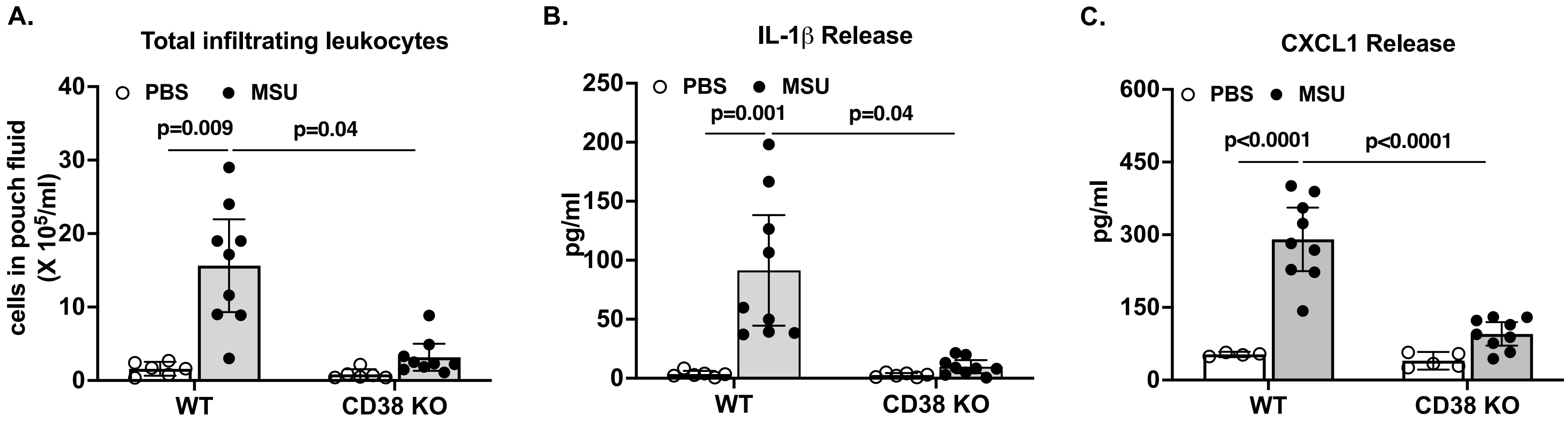

Supplemental Figure S3. CD38KO mice exhibited attenuated inflammatory responses to MSU crystals in the air pouch model *in vivo*. Air pouches were created in WT and CD38KO mice. Acute inflammatory responses to MSU crystals were determined by measuring the numbers of infiltrating leukocytes and production of IL-1β and CXCL1 in the air pouch exudate 6 hours post-injection (A-C). Data were generated with 9 biological replicates (mice) per group and expressed as mean with 95% CI. Statistical analysis was performed using Kruskal-Wallis with Dunn's multiple comparison test (for A and B) and Two-way *ANOVA* with Tukey multiple comparison test (for C).

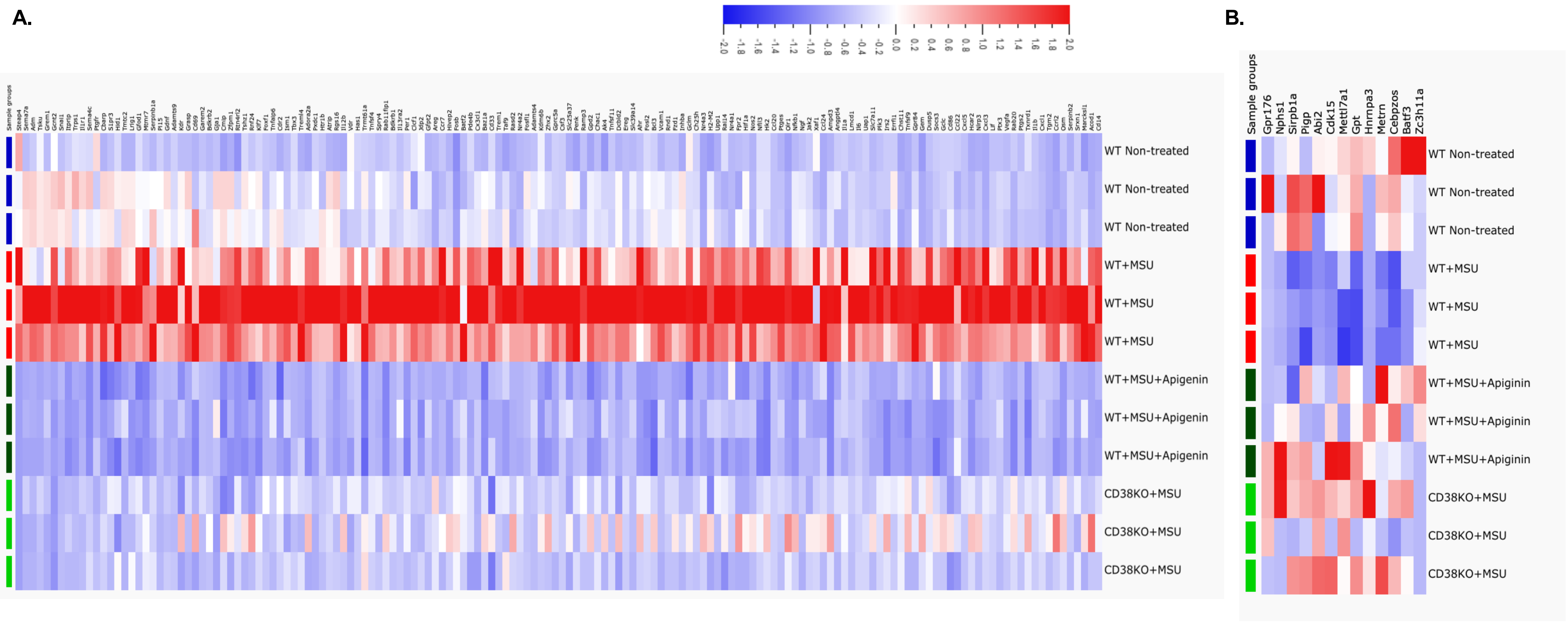

Supplemental Figure S4. Heatmaps of 154 DEGs upregulated by MSU crystals but down regulated by both apigenin and CD38 knockout (A) and of 13 DEGs downregulated by MSU crystals bot upregulated by both apigenin and CD38 knockout (B) in WT-non-treated, WT+MSU, WT+MSU+apigenin and CD38KO+MSU groups with 3 biological replicates for each group.

A.

number of nodes:	154
number of edges:	587
average node degree:	7.62
avg. local clustering coefficient:	0.454
expected number of edges:	139
PPI enrichment p-value:	< 1.0e-16

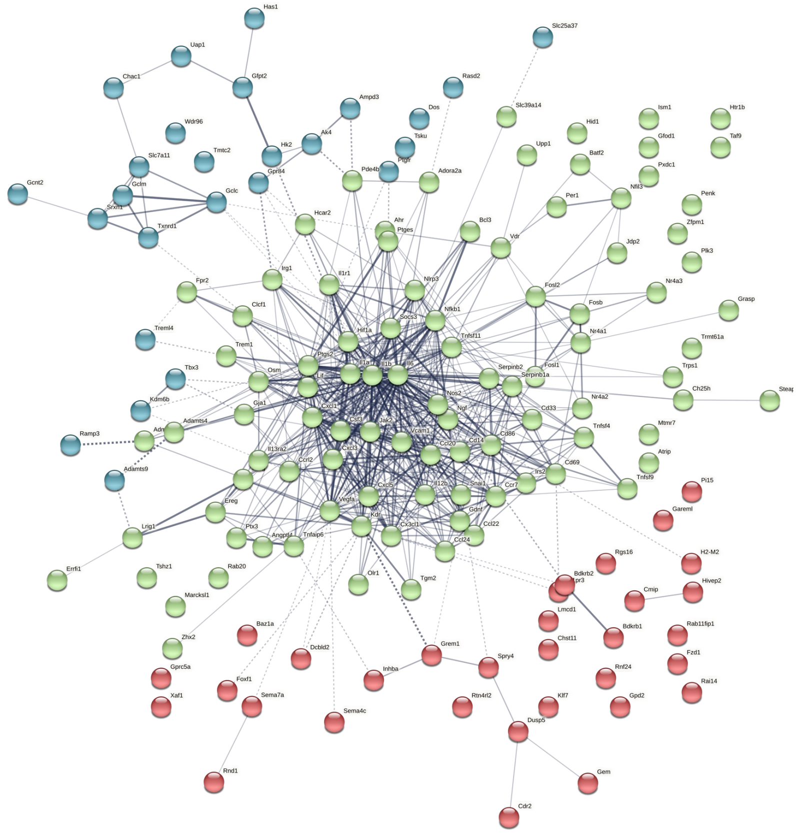

B.

Molecular Function (Gene Ontology): Green cluster				
**GO-term**	**description**	**count in network**	**strength**	**false discovery rate**
GO:0045236	CXCR chemokine receptor binding	4 of 13	1.85	0.0001
GO:0008009	Chemokine activity	7 of 41	1.59	3.65E-07
GO:0042379	Chemokine receptor binding	8 of 64	1.46	2.60E-07
GO:0004879	Nuclear receptor activity	5 of 42	1.44	2.30E-04
GO:0048020	CCR chemokine receptor binding	5 of 44	1.42	0.00026
GO:0001223	Transcription coactivator binding	3 of 26	1.42	0.0195
GO:0005125	Cytokine activity	20 of 215	1.33	1.65E-17
GO:0005164	Tumor necrosis factor receptor binding	3 of 33	1.32	3.42E-02
GO:0004896	Cytokine receptor activity	23 of 271	1.29	2.04E-19
GO:0032813	Tumor necrosis factor receptor superfamily binding	4 of 48	1.28	0.0067
GO:0001221	Transcription cofactor binding	4 of 52	1.25	8.50E-03
GO:0008083	Growth factor activity	11 of 148	1.23	3.11E-08
GO:0070851	Growth factor receptor binding	10 of 144	1.2	3.59E-07
GO:0004896	Cytokine receptor activity	5 of 93	1.09	0.0061
GO:0048018	Receptor ligand activity	25 of 475	1.08	5.29E-17

Molecular Function (Gene Ontology): Red cluster				
**GO-term**	**description**	**count in network**	**strength**	**false discovery rate**
GO:0004947	Bradykinin receptor activity	2 of 2	2.84	0.0394

Molecular Function (Gene Ontology): blue cluster				
**GO-term**	**description**	**count in network**	**strength**	**false discovery rate**
GO:0004357	Glutamate-cysteine ligase activity	2 of 2	2.93	1.35E-02

Supplemental Figure S5. 154 DEGs that were upregulated by MSU crystals but downregulated by both apigenin and CD38 knockout were subject to STRING application-Protein Query to generate a node network (A), and to Gene ontology to identify molecular function (B).

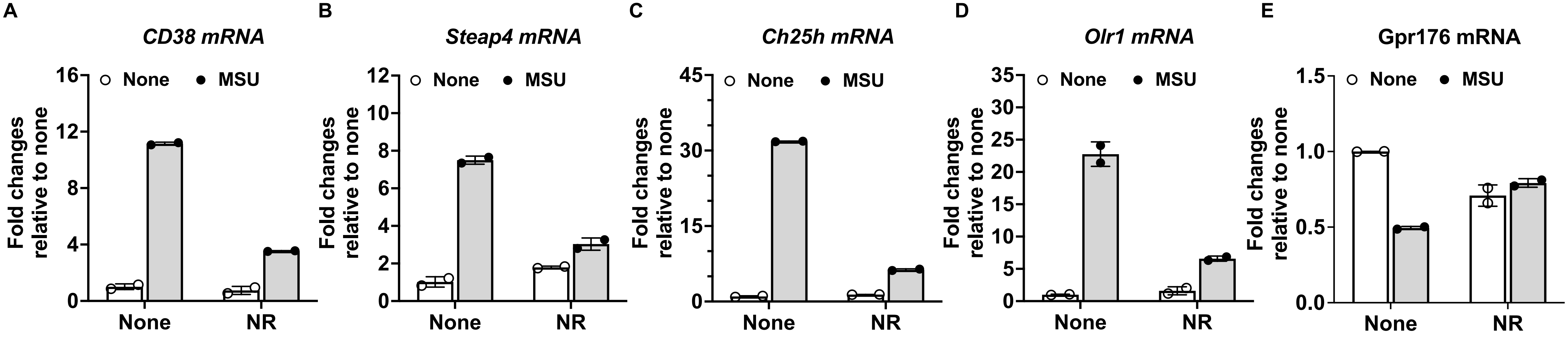

Supplemental Figure S6. Effect of NR on gene expression in BMDMs in response to MSU crystals *in vitro*. Mouse BMDMs were stimulated with MSU crystals (0.2 mg/ml) in the presence or absence of NR (0.2 mM) for 6 hours., followed by qRT-PCR analysis of expression of *CD38*, *Steap4*, *Ch25h*, *Olr1* and *Gpr176*. NR significantly inhibited MSU crystal-induced gene expression of *CD38*, *Steap4*, *Ch25h* and *Olr1* (A-D) and reversed MSU crystal-induced reduction of *Gpr176* (E).

#1	NAD	NADH	NAD/NADH	NAD/NADH (relative to none)
none	431.638	117.094	3.755	1.000
MSU	460.847	223.196	2.222	0.580
Api, 25 uM	449.577	70.091	7.349	1.896
MSU+Api	458.932	72.752	6.706	1.758
#2	NAD	NADH	NAD/NADH	NAD/NADH (relative to none)
none	483.770	111.759	4.330	1.000
MSU	519.281	227.969	2.312	0.533
Api, 25 uM	482.292	66.797	7.311	1.686
MSU+Api	481.991	80.952	5.957	1.377
#3	NAD	NADH	NAD/NADH	NAD/NADH (relative to none)
none	513.864	114.541	4.491	1.000
MSU	559.706	205.476	2.739	0.612
Api, 25 uM	495.848	73.678	6.730	1.500
MSU+Api	512.028	72.161	7.109	1.583

Supplemental Table I. Measurement of intracellular NAD^+ and NADH in BMDMs. Mouse BMDMs were stimulated with MSU crystals (0.2 mg/ml) in the presence or absence of apigenin (25 uM) for 24 hours. The cellular NAD+ and NADH content were measured using a NAD^+/NADH quantification colorimetric kit (BioVision), and the NAD/NADH ratio was determined. The fold changes relative to none (non-treated) were calculated.

Measuring NAD^+ levels from whole blood

To 100µl blood samples, 400µl -80°C methanol containing mass + 3 NAD^+ (45.5µM) was added and sonicated at 4°C 3x for 10 seconds each. Following addition of 400µl $CHCl_3$ and vortexing, 200µl H_2O was added and samples were vortexed well. Samples were then centrifuged at 13,000rpm for 15 minutes at 4°C. 400µl of the top aqueous phase containing the metabolites was transferred to a clean 1.5mL Eppendorf, flash-frozen at -80°C using liquid nitrogen and subjected to lyophilization. Samples were reconstituted in 20µl degassed buffer A (10mM ammonium acetate in H_2O + 0.1% formic acid), centrifuged 13,000rpm for 15 minutes at 4°C, and transferred to mass spectrometry tubes for analysis.

An Agilent 1200 Series HPLC coupled to a Thermo Q Exactive Plus mass spectrometer was used for sample analysis. An autosampler was used to inject the samples onto an Agilent Zorbax 300SB-C18 reversed-phase column (2.1 × 150 mm, 5-micron) using a 1.0 µL injection volume for each. Solvent A consisted of 10 mM ammonium acetate in H_2O + 0.1% formic acid, and Solvent B consisted of methanol + 0.1% formic Acid. A flow rate of 50 µL per minute was used for the first 6 minutes of the run at 2.0% B solvent, followed by a gradient from 2.0–95.0% B from 6 to 20 minutes along with an increased flow rate of 150 µl per minute. The solvent was held at 95.0% B from 20 to 30 min and returned to 2.0% B for the remainder of the run. A HESI (heated electrospray ionization) source was used with positive polarity, a capillary temperature of 320 °C, the source voltage of 3.2 kV, S-lens RF level of 60, and a sheath gas flow rate of 16.0. One full scan from 100–900 *m/z* was performed at 17,500 resolution, followed by targeted MS^2 scans of NAD M+3 (667.1 *m/z*) and NAD^+ (664.1 *m/z*) at 35,000 resolution with an isolation width of 2.0 *m/z*. Normalized CID collision energy was set to 25.
